# Emergence of KPC-producing *Klebsiella pneumoniae* in Uruguay: infection control and molecular characterization

**DOI:** 10.1002/nmi2.40

**Published:** 2014-04-04

**Authors:** C Marquez, A Ingold, N Echeverría, A Acevedo, R Vignoli, V García-Fulgueiras, J Viroga, O Gonzalez, V Odizzio, K Etulain, E Nuñez, H Albornoz, G Borthagaray, A Galiana

**Affiliations:** 1 Cátedra de Microbiología, Instituto de Química Biológica, Facultad de Ciencias y de Química-UdelaR, Universidad de la República Montevideo, Uruguay; 2 Facultad de Química-UdelaR, Cátedra de Microbiología, UdelaR Montevideo, Uruguay; 3 Dpto Bacteriología y Virología, Instituto de Higiene/Facultad de Medicina-UdelaR Montevideo, Uruguay; 4 Laboratorio Gram/Microbiología, Sanatorio SemmMautone Maldonado, Uruguay; 5 Comité de Infecciones, Sanatorio SemmMautone Maldonado, Uruguay; 6 Unidad de Cuidados Intensivos, Sanatorio SemmMautone Maldonado, Uruguay; 7 Unidad de Cuidados Intensivos, Hospital Maciel Montevideo, Uruguay; 8 Facultad de Química-UdelaR, Bioquímica Clínica Montevideo, Uruguay; 9 Dpto Microbiología, Hospital Maciel, UDYCI Montevideo, Uruguay

**Keywords:** Antibiotic resistance, infection control, KPC

## Abstract

We describe the first outbreak of *Klebsiella pneumoniae* carbapenemase-producing *K. pneumoniae* (KPC-KP), the infection control measures adopted and the shift in resistance patterns of isolates during antibiotic treatment. The ST258 KPC-KP strain exhibited a multiresistant antibiotic phenotype including co-resistance to gentamycin, colistin and tigecycline intermediate susceptibility. Isolates before and after treatment had different behaviour concerning their antibiotic susceptibility and the population analysis profile study. A progressive increase in the aminoglycosides (acquiring amicacin resistance) and β-lactam MICs, and a decreased susceptibility to fosfomycin was observed throughout the administration of combined antimicrobial regimens including meropenem. A high meropenem resistance KPC-KP homogeneous population (MIC 256 Jg/mL), could arise from the meropenem heterogeneous low-level resistance KPC-KP population (MIC 8 Jg/mL), by the selective pressure of the prolonged meropenem therapy. The *kpc* gene was inserted in a Tn*4401* isoform a, and no transconjugants were detected. The core measures adopted were successful to prevent evolution towards resistance dissemination.

## Introduction

Carbapenems are recommended for severe infections caused by extended spectrum *β*-lactamase (ESBL) -producing Enterobacteria, because of their resistance to third-generation cephalosporins and associated resistance to almost all other antibiotics. Therefore the emergence of carbapenem-resistance in Enterobacteria is worrying, and could be related to several combined mechanisms involving outer membrane permeability defects, hyperproduction of AmpC cephalosporinases, ESBL and carbapenemase production 1.

*Klebsiella pneumoniae* carbapenemases (KPC) are the most frequent carbapenemases found in *Klebsiella pneumoniae* and are mostly plasmid encoded, but chromosomal location has also been reported 2. As the gene encoding KPC usually resides on transferable plasmids, it is capable of disseminating to other Gram-negative genera 3. The worldwide spread of KPC-producing *K. pneumoniae* strains (KPC-KP) has revealed the successful dissemination of a major clone defined as sequence type 258 (ST258). Since 2006, KPC-KP has arisen in South America, particularly in countries bordering Uruguay—Argentina and Brazil 4,5.

A high frequency of ESBL-producing *K. pneumoniae* isolates (70%) has been reported in intensive care unit (ICU) patients but no cases of KPC-producing isolates had been reported in the country up to April 2011 6. We describe the first two clinical cases of KPC-KP infections in Uruguay, their phenotypic and genotypic characteristics, the shift of resistance patterns during antibiotic treatment and the infection control measures adopted.

## Methods

### Hospital setting characteristics

The ‘Sanatorio Mautone’ is a 65-bed acute general hospital located in Maldonado-Punta del Este, a tourist area receiving 500 000 tourists annually from Argentina, Brazil and to a lesser extent from the USA and Europe. The ICU has six beds, one in an isolation room and the others in a common area separated by curtains.

### Clinical cases

#### Patient A

On 20 February 2011 a 30-year-old man (Table1) was admitted from home to the ICU for severe enteric sepsis caused by a *Salmonella* sp. that was nalidixic acid and ciprofloxacin susceptible, recovered from blood and stool cultures. He was mechanically ventilated due to septic shock and acute respiratory distress syndrome and intravenous ciprofloxacin treatment was started. This patient had Evans syndrome associated with systemic lupus erythematosus treated with corticosteroids and immunosuppressants. He had never required medical care in any other country. He first developed an *Escherichia coli* tracheobronchitis treated with piperacillin-tazobactam, and later developed a *Pseudomonas aeruginosa* ventilator-associated pneumonia with secondary bacteraemia that was treated with imipenem for 13 days. On 26 March a urine specimen grew *P. aeruginosa* and KPC-KP (A1). A second urine specimen grew KPC-KP (A2) as well. This catheter-related asymptomatic bacteriuria was not treated. On 31 March the patient developed fever and septic shock and blood cultures grew *Salmonella* sp. susceptible to nalidixic acid, ciprofloxacin and imipenem, treated with ciprofloxacin and imipenem; he progressed to refractory septic shock and died on 7 April. On 6 April the central venous catheter tip grew 10 CFU of KPC-KP (A3) and the blood cultures were negative.

**Table 1:** Characteristis of the patients, treatments and KPC-producing *Klebsiella pneumoniae* isolates

	Diagnosis and antimicrobial treatment	Date	Isolate (Specimen)	Synergy tests/*bla*_KPC-2_	Sequence type	PFGE (*Xba*I)
Patient A	SLE, Enteric *Salmonella* sepsis; CIP	20/02				
	*Escherichia coli* Tracheobronchitis; PTZ	27/02				
	*Pseudomonas aeruginosa* VAP with secondary bacteraemia; IMP	10 al 23/03				
	*P. aeruginosa* persistent tracheal	26/03	A1 (Urine)	+/nd	nd	nd
	*Samonella* bacteraemia and Septic shock; IMP plus CIP	30/03 31/03	A2 (Urine)	+/+	ST258	A
		06/04	A3 (Catheter)	+/nd	nd	nd
Patient B	Chest trauma, respiratory failure, Diabetic; PTZ	15/03				
	*P. aeruginosa* VAP with secondary bacteraemia; IMP	20 al 30/03				
	*P. aeruginosa* persistent tracheal infection	1 al 09/04				
	Mechanical ventilation reinstalled	07/04	B1 (TA) B2 (Catheter)	+/+ +/+	ST258 nd	A A
	KPC-KP VAP, Septic shock; DOX plus RIF plus MEM	09/04	B3 (TA) B4 (Urine) B5 (Rectal) B6 (TA)	+/nd +/nd +/nd +/+	nd nd nd ST258	nd nd nd A
	TIG plus RIF plus MEM Respiratory failure, *P. aeruginosa* bacteraemia	14/04 28/04				

CIP, ciprofloxacin; PFGE, pulsed-field gel electrophoresis; PTZ, piperacillin-tazobactam; IPM, imipenem; DOX, doxycycline; RIF, rifampin; MEM, meropenem; TYG, tigecycline; A_1_, >10^5^ CFU/mL *K. pneumoniae* and *P. aeruginosa*; A_2_, >10^5^ CFU/mL of *K. pneumoniae*; A_3_, 10 CFU of *K. pneumoniae* in the tip of central line catheter; B_2_, 100 CFU of *K. pneumoniae*; B_4_, >10^5^ CFU mL^−1^ of *K. pneumoniae*; +, difference of ≥5 mm in zones between MEM and MEM plus boronic acid and <5 mm in zone between MEM and MEM plus cloxacillin and MEM plus dipicolinic acid; nd, not determined; SLE, systemic lupus erythematosus; VAP, ventilator-associated pneumonia; TA, tracheal aspirate.

#### Patient B

On 15 March 2011 a 67-year-old man (Table1) with severe chest trauma was hospitalized in ICU with respiratory failure requiring mechanical ventilation. Piperacillin-tazobactam treatment was started for suspected pulmonary infection. The patient was diabetic with a history of chronic hypertension. He had not required medical care in any other country. On 20 March the patient developed a *P. aeruginosa* ventilator-associated pneumonia with secondary bacteraemia treated with imipenem for 9 days and then with piperacillin-tazobactam and ciprofloxacin. Patient condition improved and the orotracheal tube was removed on 4 April. Three days later he developed respiratory failure and mechanical ventilation was reinstalled. On 7 April KPC-KP (B1) was recovered from respiratory secretions and from the central venous catheter tip (100 CFU) (B2). On 9 April the patient developed a ventilator-associated pneumonia caused by KPC-KP (B3) with septic shock. Blood cultures were negative, and cultures from urine and perianal swabs grew KPC-KP (B4, B5). Initially a high dose of doxycycline (200 mg every 6 h) plus rifampicin (600 mg every 24 h) and meropenem (2 g in a 3-h infusion every 8 h) were started. The patient's condition improved and noradrenalin infusion was stopped. On 14 April doxycycline was substituted by tigecycline (100 mg every 12 h), which became available for the first time in Uruguay. Antimicrobial treatment was stopped on 25 April. On 28 April the respiratory failure persisted, respiratory secretions were positive for KPC-KP (B6) and *P. aeruginosa* and blood culture was positive for *P. aeruginosa*. Finally on 3 May the patient died of refractory respiratory failure.

### Infection prevention and control measures

The Infection Control Committee, considering the unusual antimicrobial resistance phenotype of *K. pneumoniae* isolates suspected nosocomial cross-transmission and decided to implement control measures, perform an epidemiological investigation and notify the public health authorities 7,8. Control measures adopted were: meeting with hospital (clinicians, nurses, laboratory, housekeeping) and administration staff; extended contact isolation precautions for ICU patients with daily monitoring (isolation room and dedicated staff for the colonized and the infected patients, exclusive work clothes to use with these patients, strict hand hygiene with alcohol gel, reinforcement of the unshared use of instruments and disinfection between patients of shared instruments); screening of patients in contact with colonized or infected patients and pre-emptive isolation while awaiting results; closing the ICU to new patients; and reinforcement of environmental hygiene with alcohol or chlorinated agents. The surveillance cultures for the identification of KPC-KP intestinal-tract-colonized patients were performed according to the CDC protocol 9. Respiratory secretions and urine were evaluated following conventional culture methods.

### Identification and antimicrobial susceptibility studies

The identification and the antibiotic susceptibility testing were performed using the VITEK 2 C (bioMérieux, Marcy l'Etoile, France) system. The MICs of imipenem, meropenem, tigecycline, colistin and gentamycin were determined using *E*-test (bioMérieux). CLSI clinical breakpoints were applied, except for tigecycline and colistin where EUCAST (http://www.eucast.org) breakpoints were used 10,11. ESBL testing was performed according to CLSI guidelines.

Susceptibility to fosfomycin was assayed by disc diffusion 12. KPC phenotype was determined by synergy test using KPC/MBL confirm kit (ROSCO Diagnostica A/S, Denmark). The population analysis profile was determined for meropenem as described elsewhere 13.

### Molecular studies

Screening for resistance genes was performed by PCR, following reported conditions for *bla*_TEM_, *bla*_SHV_, *bla*_CTX_-_M_, *bla*_KPC_, *bla*_IMP_, *bla*_VIM_ gene families, and *bla*_PER-2_, *aacA4*, *qnrA* and *qnrB* genes 14–20.

The genetic environment of *bla*_KPC-2_ gene was studied by PCR mapping with specific primers for IS*Kpn6* or IS*Kpn7* or *tnpA* and *bla*_KPC-2_. PCR amplification of the complete *ompK36* gene was performed 21.

Crude extracts were subjected to isoelectrofocusing with the use of PhastSystem and PhastaGels IEF 3–9 (Pharmacia, Stockholm, Sweden).

### Genetic relatedness

The genetic relatedness of KPC-KP isolates was evaluated by pulsed field gel electrophoresis using *Xba*I-digested total DNA and interpreted by Tenover criteria 22. Multilocus sequence typing with seven housekeeping genes was performed according to the protocol described on the *K. pneumoniae* multilocus sequence typing website (http://www.pasteur.fr/mlst/Kpneumoniae.html).

### Mating experiments

To determine whether the *bla*_KPC-2_ gene was on a transferable element, isolates A1 and B1—cefotaxime resistant, rifampin and sodium azide sensitive—were mated in three independent assays with *E. coli* strain Top10—cefotaxime susceptible, rifampin and sodium azide resistant. They were cultured for 18 h at 35°C, in Müller–Hinton agar (MHA) containing cefotaxime 2 mg/L or rifampin 200 mg/L. The growth was suspended in MH broth (MHB) to achieve a 1 MacFarland turbidity standard, and 0.5 mL of the mix of equal volumes of the donor and receptor suspensions was plated as a drop in the middle of an MHA plate, and incubated for 18 h at 35°C. Serial decimal dilutions of the suspension in 1 mL MHB of the total growth were prepared. Transconjugants were selected from 10^−1^ and 10^−2^ dilutions in MHA containing cefotaxime 2 mg/L and rifampin 200 mg/L and confirmed by positive growth in MHA containing sodium azide 100 μg/mL and positive PCR for *bla*_KPC-2_ gene.

## Results

The ICU remained closed to new admissions while KPC-KP-infected patients were still hospitalized. Three patients were considered contacts because they shared their stay in ICU with KPC-KP-infected patients. Two of them were discharged with negative surveillance cultures that persisted negative on days 15 and 30 thereafter. Surveillance cultures of the third contact were performed every 48 h with negative results. The epidemiological investigation identified a diabetic patient with a history of contact with the healthcare system in New York, USA, who was admitted to the ICU 17 days before the first isolation of KPC-KP. She had a severe urosepsis caused by non-multiresistant *E. coli* and had multiple episodes of diarrhoea during the 5 days of hospitalization. When the ICU was free of patients, it underwent a thorough cleaning and environmental disinfection. Five months later two colonized cases were detected (asymptomatic urinary catheter bacteriuria and hepatic drain of quistostomy), similar control measures were applied, surveillance cultures were performed during the next 6 months and no more cases have been identified since then.

All *K. pneumoniae* isolates recovered showed a multiresistant phenotype but were susceptible to fosfomycin, ESBL-negative and exhibited a KPC phenotype. Different values for MICs to *β*-lactams and gentamycin were observed for the isolates from patient B (Table2). The fosfomycin inhibition zone diameters were 22 and 17 mm for B1 and B6 isolates, respectively. Scattered colonies were observed within the inhibition zone of meropenem for A2 and B1 whereas B6 showed no inhibition zone.

**Table 2:** Susceptibility of KPC-producing *Klebsiella pneumoniae* isolates

	Vitek MIC μg mL^−1^ to:											*E*-test MIC μg mL^−1^ to:			
*K. pneumoniae* isolate	TZP	FOX	CTX	CAZ	CEP	GEM	ANK	CIP	COL	IPM	MEM	IPM	MEM	TYG	COL
A1	≥128/4	≥64	16	16	8	≥16	16	≥4	≥16	8	4	8	4	2	24
A2	≥128/4	≥64	16	16	8	≥16	16	≥4	≥16	8	4	8	4	2	24
B1	≥128/4	≥64	16	16	8	≥16^a^	32	≥4	≥16	≥16	4	8	8	2	24
B2	≥128/4	≥64	16	16	8	≥16	32	≥4	≥16	≥16	4	8	8	2	24
B3	≥128/4	≥64	16	16	8	≥16	32	≥4	≥16	≥16	4	8	8	2	24
B5	≥128/4	≥64	16	32	8	≥16	32	≥4	≥16	≥16	8	16	8	2	24
B6	≥128/4	≥64	≥64	≥64	≥64	≥16^b^	≥64	≥4	≥16	≥16	≥16	≥32	≥32^c^	2	24

PTZ, piperacillin-tazobactam; FOX, cefoxitin; CTX, cefotaxime; CAZ, ceftazidime; CEP, cefepime; GEM, gentamicin; ANK, amikacin; CIP, ciprofloxacin; COL, colistin; IPM, imipenem; MEM, meropenem; TYG, tigecycline.

a *E*-test MIC 36 μg/mL.

b *E*-test MIC 96 μg/mL.

c *E*-test MIC 256 μg/mL.

The B1 isolate population analysis profile showed a heterogeneous population with a subset of 0.01–0.001% growing at meropenem concentrations ranging from 16 to 128 µg/mL. The B6 isolate population analysis profile showed that >0.01% of cells grew up to 512 µg/mL.

**Figure 1:**
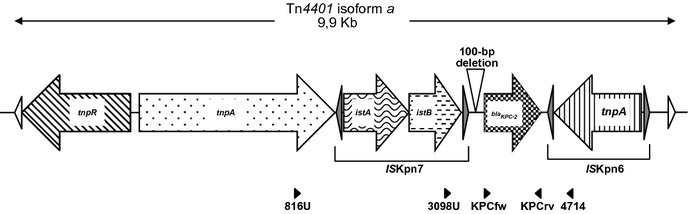
Schematic representation of the genetic structures from Tn*4401* isoform *a* identified in the surroundings of *bla*_KPC-2_ gene. Genes and their corresponding transcription orientations are shown by horizontal arrows. Empty triangles represent inverted repeats (IRs) that flank Tn*4401* structure. Internal grey triangles represent IRs from *IS*Kpn6 and *IS*Kpn7. The 100-bp deletion characteristic of Tn*4401* isoform *a* is indicated above the sequence. Primers used are shown below the sequence: KPCfw, KPCrv 15, 816U, 3098U, 4714 26.

The isolates A2, B1, B2 and B6 revealed the presence of a *bla*_KPC-2_, *bla*_TEM-1_, *bla*_SHV-1_ and *aacA4* genes. Isoelectrofocusing showed that these isolates shared two common β-lactamase activities with isoelectric points of 5.4 and 6.7, characteristic of TEM-1 and KPC-2 enzymes, respectively. PCR mapping revealed a 4-kb genetic region containing *tnp*A, IS*Kpn7*, *bla*_KPC-2_ and IS*Kpn6* genes, consistent with a Tn*3*-based Tn*4401* isoform a, since a 100-bp deletion was detected upstream of the *bla*_KPC-2_ gene (Fig.1) 23. Sequence analysis of *ompK36* DNA revealed a 99% similarity with a described gene encoding a functional OmpK36 porin (Accession number FJ577673).

Biparental conjugation did not render transconjugants, indicating a conjugation frequency less than 1 × 10^−8^/recipient.

## Discussion

In the present study we describe the first nosocomial outbreak caused by KPC-KP in Uruguay. Considering that none of the patients had a history of recent travel abroad, it is possible that an unrecognized colonized patient with a history of admission in a healthcare facility in one of the endemic areas introduced the causative agent to our hospital, as may be the case identified in the root-cause analysis 24.

The KPC-KP isolates exhibited a multiresistant antibiotic phenotype including resistance to colistin and intermediate susceptibility to tigecycline. Based on pulsed field gel electrophoresis analysis they represent a single clone and they belong to the internationally distributed ST258 KPC-KP clone, allowing us to propose their introduction and the occurrence of cross-transmission between patients. Both clinical cases had risk factors previously reported to be associated with the acquisition of KPC-producing bacteria: prolonged ICU stay, invasive devices, immunosuppression and previous multiple antibiotic therapies, including ciprofloxacin, tazobactam-piperacillin and imipenem 25.

This is the first description of isolates belonging to the KPC-KP ST258, exhibiting co-resistance to gentamycin, colistin and tigecycline intermediate susceptibility, acquiring further amikacin resistance.

The first patient had a KPC-KP asymptomatic catheter-related bacteriuria, considered the source for the cross-transmission, and his death was related to *Salmonella* sepsis. In contrast, the second patient had multiple sites colonized, and developed severe sepsis associated with KPC-KP ventilator-associated pneumonia.

The core measures adopted according to European expert opinion were reinforced and have been successful in preventing evolution towards resistance dissemination 8.

The deletions in the upstream genetic environment of the *bla*_KPC_ gene increased the level of KPC production, in accordance with the high-level of carbapenem resistance observed 26. Even though B1 and B6 isolates were the same strain based on pulsed field gel electrophoresis analysis, they had different behaviours concerning their antibiotic susceptibility and the population analysis profile study. A progressive increase in the MICS for aminoglycosides and β-lactams, and a decreased susceptibility to fosfomycin was observed throughout the administration of combined antimicrobial regimens in patient B. Even though pre- and post-treatment isolates possessed *ompk36* genes identical to a previously described functional protein, a decrease in the *ompk36* expression level could not be discounted.

In addition, selection of mutants with resistance to different antibiotic classes could have emerged by the acquisition of a resistance mechanism with a broad range specificity spectrum. Further studies are needed to understand the molecular basis of these complex and variable resistance phenotypes and to evaluate their frequency and clinical significance. To counteract the emergency of carbapenem resistance, new antibiotic regimens need to be developed and hospital infection control and diagnostic microbiology practice must be improved.

## Conflict of Interest

None declared.
